# Impact of Non-HIV and HIV Risk Factors on Survival in HIV-Infected Patients on HAART: A Population-Based Nationwide Cohort Study

**DOI:** 10.1371/journal.pone.0022698

**Published:** 2011-07-25

**Authors:** Niels Obel, Lars Haukali Omland, Gitte Kronborg, Carsten S. Larsen, Court Pedersen, Gitte Pedersen, Henrik Toft Sørensen, Jan Gerstoft

**Affiliations:** 1 Department of Infectious Diseases, Copenhagen University Hospital, Rigshospitalet, Copenhagen, Denmark; 2 Department of Infectious Diseases, Copenhagen University Hospital, Hvidovre Hospital, Hvidovre, Denmark; 3 Department of Infectious Diseases, Aarhus University Hospital, Aarhus Sygehus, Aarhus N, Denmark; 4 Department of Infectious Diseases, Odense University Hospital, Odense C, Denmark; 5 Department of Infectious Diseases, Aarhus University Hospital, Aalborg Sygehus, Aalborg, Denmark; 6 Department of Clinical Epidemiology, Aarhus University Hospital, Aarhus Sygehus, Aarhus N, Denmark; 7 Department of Epidemiology, Boston University School of Public Health, Boston, Massachusetts, United States of America; University of Cape Town, South Africa

## Abstract

**Background:**

We determined the impact of three factors on mortality in HIV-infected patients who had been on highly active antiretroviral therapy (HAART) for at least one year: (1) insufficient response to (HAART) and presence of AIDS-defining diseases, (2) comorbidity, and (3) drug and alcohol abuse and compared the mortality to that of the general population.

**Methodology/Principal Findings:**

In a Danish nationwide, population-based cohort study, we used population based registries to identify (1) all Danish HIV-infected patients who started HAART in the period 1 January 1998–1 July 2009, and (2) a comparison cohort of individuals matched on date of birth and gender (N = 2,267 and 9,068, respectively). Study inclusion began 1 year after start of HAART. Patients were categorised hierarchically in four groups according to the three risk factors, which were identified before study inclusion. The main outcome measure was probability of survival from age 25 to 65 years. The probability of survival from age 25 to age 65 was substantially lower in HIV patients [0.48 (95% confidence interval (CI) 0.42–0.55)] compared to the comparison cohort [0.88 (0.86 to 0.90)]. However, in HIV patients with no risk factors (N = 871) the probability of survival was equivalent to that of the general population [0.86 (95% CI 0.77–0.92)]. In contrast, the probability of survival was 0.58 in patients with HIV risk factors (N = 704), 0.30 in patients with comorbidities (N = 479), and 0.03 in patients with drug or alcohol abuse (N = 313).

**Conclusions:**

The increased risk of death in HIV-infected individuals is mainly attributable to risk factors that can be identified prior to or in the initial period of antiretroviral treatment. Mortality in patients without risk factors on a successful HAART is almost identical to that of the non–HIV-infected population.

## Introduction

Since the introduction of highly active antiretroviral therapy (HAART), the risk of death has decreased substantially in the HIV-infected population, but remains markedly higher than in the general population [1]. Several explanations have been proposed, including the detrimental impact of non-HAART-related lifestyle factors such as smoking and drug abuse, presence of other diseases, exposure to co-pathogens (*e.g.*, hepatitis C), side effects of HAART (*e.g.*, myocardial infarction), presence of HIV-related diseases, and failure to obtain full immune reconstitution [2]–[6].

Recently it has been proposed that, despite successful treatment, HIV-infected patients suffer from accelerated aging driven by residual immune activation [7]. Limitations of previous research on risk of death among HIV-infected individuals include non-population based designs, lack of access to information on major pre-HIV-infection and post-HAART risk factors, as well as lack of information on risk factors in general population comparison cohorts [8]. Three groups of risk factors consistently have been reported to increase mortality among the HIV-infected patients on HAART: (1) HIV-related risk factors (AIDS defining diseases and insufficient response to HAART), (2) comorbidities including hepatitis C, and (3) drug and alcohol abuse.

We used a Danish population-based cohort of HIV-infected patients and a comparison cohort from the general population to (1) estimate the impact of risk factors identifiable in the initial period of antiretroviral therapy on long-term mortality in the HIV-infected population and (2) determine the relative risk of death, compared to the background population, among successfully treated HIV-infected patients without such risk factors.

## Methods

### Ethics

Since the study is entirely based on data from national registries and clinical databases, there was according to Danish law no request for an ethical permission (Denmark has no Institutional review boards). The study was approved by Danish Data Protection Agency.

### Setting

Among Denmark's population of 5.4 million, the estimated prevalence of HIV-infection in adults is 0.09%. Denmark's tax-funded health care system provides antiretroviral treatment free-of-charge to all HIV-positive residents. During the study period, national guidelines stipulated one or more of the following criteria for initiating HAART in HIV-positive individuals: presence of an HIV-related disease, acute HIV-infection, pregnancy, CD4 cell count <300 cells/µl, and, until 2001, plasma HIV-RNA>100,000 copies/ml. Patients are seen on an outpatient basis at intended intervals of 12 weeks.

### Data Sources

We used the unique 10-digit Central Person Registration (CPR) number assigned to all Danish citizens at birth or immigration, to avoid multiple registrations and to track individuals in national healthcare registries. HIV-infected patients were identified from the Danish HIV Cohort Study, which includes all HIV-infected patients treated in Denmark's eight specialized HIV centers since January 1, 1995 [9]. The Danish Civil Registration System (CRS), a national registry containing information on the vital status of all citizens and other demographic data [10] allowed us to identify a population-based comparison cohort and to extract data on birth, gender, date of immigration and emigration, loss to follow-up, date of death, and place of birth, both for the HIV-infected patients and the comparison cohort. Data on comorbidity, alcohol abuse, and drug abuse were extracted from the Danish National Registry of Patients, which contains information on all patients discharged from Danish non-psychiatric hospitals [11]. Records for each inpatient admission include dates of admission and discharge and diagnoses, coded by the attending physician according to the *International Classification of Diseases*.

### Study populations

We included HIV-infected patients aged 25–65 who started HAART between 1 January 1998 and 1 July 2009, were registered in the CRS, and lived in Denmark at start of HAART. HAART was defined as a treatment regimen of at least three antiretroviral drugs or a treatment regimen including a combination of a non-nucleoside reverse transcriptase inhibitor and a boosted protease inhibitor. For HIV-infected patients, the index date was defined as 1 year after start of HAART.

We also used the CRS to identify a comparison cohort drawn from the general population, consisting of 4 individuals for each HIV-infected patient, matched on age and date of birth. To be eligible, matched individuals had to be alive on the index date of the corresponding HIV-infected patient, had to be living in Denmark on the date the corresponding patient started HAART, and could not be registered as having a comorbidity included in the Charlson Comorbidity Index (CCI), alcoholism, or drug abuse, as of the index date. The index date of individuals in the comparison cohort was defined as the index date of the corresponding HIV-infected patient.

### Risk factors

Risk factors were defined as follows:

#### HIV risk factors

Detectable viral load (>49 copies/ml) and/or CD4 below 200 cells/ul at the last measurement prior to the index date and/or AIDS- defining disease as of the index date.

#### Comorbidity

Diagnosed with comorbidity (excluding AIDS-defining diseases) as defined in the Charlson comorbidity index (CCI) [12]–[14] before the index date. Hepatitis C, if registered in the Danish HIV Cohort Study, also was included as comorbidity.

#### Alcohol and drug abuse

Drug abuse reported as the route of HIV transmission or hospital contacts before the index date with the following diagnoses: ICD-8: 29100–29199, 57109, 57110, 30300–30389, 30391–30399, 30409–30499; and ICD-10: K700–K709, F102–109, G312, F110–199, or T400.

### Statistical analysis

For HIV-infected patients and the comparison cohort, we calculated person-years (PY) of follow-up from the index date to age 65, emigration, date of death, or 1 July 2010, whichever came first. The study outcome was time to death from any cause.

Study participants were categorized into the following risk groups:

Group 0 (General population comparison cohort): HIV risk factors: −, comorbidity: −, abuse: −.

Group 1 (HIV-infected patients): HIV risk factors: −, comorbidity: −, abuse: −.

Group 2 (HIV-infected patients): HIV risk factors: +, comorbidity: −, abuse: −.

Group 3 (HIV-infected patients): HIV risk factors: +/−, comorbidity: +, abuse: −.

Group 4 (HIV-infected patients): HIV risk factors: +/−, comorbidity: +/−, abuse: +..

We computed Kaplan-Meier tables using age as the time variable and stratifying on the above 5 risk groups. We calculated mortality rate ratios (MMRs) and corresponding 95% confidence intervals for each risk group, stratified into two age categories (25–<45 years and 45–65 years) [15]. In stratified analyses, only individuals in the population comparison cohort who were matched to the respective HIV patients were included in the calculations.

SPSS version 15.0 (SPSS Inc., Chicago, Il, USA) and Stata version 8.0 (Stata Corporation, College Station, Texas, USA) were used to perform the analyses.

## Results

We identified 871 individuals in Group 1, 704 in Group 2, 379 in Group 3, 313 in Group 4, and 9,068 in the general population comparison cohort (Group 0), which in stratified analyses were divided in 4 comparison cohorts (for Group 1, 3484 individuals, Group 2, 2816 individuals, Group 3, 1516 and Group 4, 1252 individuals). Due to the inclusion criteria, no individuals in the general population comparison cohort were diagnosed with comorbidity or drug or alcohol abuse as of the index date (Table 1). The major comorbidity in the Charlson index was attributable to liver diseases (table 1). More HIV-infected patients were born outside Denmark than persons in the comparison cohort. Other characteristics of the study cohorts are described in Table 1.

**Table 1:** Characteristics of the study population.

	General population comparison cohort(Group 0)	HIV-infected patients with no risk factors(Group 1)	HIV-infected patients with HIV risk factors(Group 2)	HIV-infected patients with comorbidity(Group 3)	HIV-infected patients with alcohol/drug abuse(Group 4)
Number of patients	9,068	871	704	379	313
Median years of follow up, years	5.91	5.15	6.35	4.60	5.01
Person-years of follow up (years)	53,015	4,614	4,296	1,858	1,599
Median age at study entry, years (IQR*)	40 (35–47)	40 (34–46)	39 (34–46)	44 (38–53)	40 (36–46)
Males (n, %)	6,704 (73.9%)	659 (75.7%)	493 (70.0%)	318 (83.9%)	206 (65.8%)
Deceased during follow-up (n, %)	138 (1.5%)	15 (1.7%)	51 (7.2%)	58 (15.0%)	89 (28.4%)
Emigrated during follow-up (n, %)	91 (1.0%)	16 (1.80%)	21 (4.0%)	7 (1.8%)	1 (0.6%)
Lost to follow up (n, %)	2 (0.0%)	3 (0.3%)	2 (0.3%)	2 (0.5%)	1 (0.3%)
Born outside Denmark (n, %)	677 (7.5%)	253 (29.0%)	276 (39.2%)	90 (23.7%)	45 (14.4%)
Comorbidity (n, %)**	0 (0%)	0 (0%)	0 (0%)	379 (100.0%)	262 (83.7%)
Neurologic disease (n, %)	0 (0%)	0 (0%)	0 (0%)	50 (13.2%)	10 (3.27%)
Cardiovascular disease (n, %)	0 (0%)	0 (0%)	0 (0%)	30 (7.9%)	16 (5.1%)
Pulmonay disese(n, %)	0 (%)	0 (0%)	0 (0%)	56 (14.8%)	27 (8.6%)
Gastroentorologic disease (n, %)	0 (%)	0 (0%)	0 (0%)	42 (11.1%)	18 (5.8%)
Liver disease (n, %)	0 (%)	0 (0%)	0 (0%)	148 (39.1%)	251 (80.2%)
Renal disease (n, %)	0 (%)	0 (0%)	0 (0%)	17 (3.9%)	3 (1.0%)
Diabetes (n, %)	0 (%)	0 (0%)	0 (0%)	22 (5.8%)	4 (1.3%)
Rheumatological disease (n, %)	0 (%)	0 (0%)	0 (0%)	17 (4.5%)	1 (0.3%)
Malignant disease (n, %)	0 (%)	0 (0%)	0 (0%)	62 (16.4%)	10 (3.2%)
Abuse (n, %)	0 (0%)	0 (0%)	0 (0%)	0 (0%)	323 (100.0%)
Drug abuse(n, %)	0 (%)	0 (0%)	0 (0%)	0 (0%)	266 (85.0%)
Alcohol abuse (n, %)	0 (%)	0 (0%)	0 (0%)	0 (0%)	93 (29.7%)
Hepatitis C (n, %)	0 (%)	0 (0%)	0 (0%)	107 (28.2%)	240 (76.7%)
Infection mode:					
Homosexual contact (n, %)		454 (52.1%)	303 (43.0%)	194 (51.2%)	24 (7.7%)
Heterosexual contact (n, %)		379 (43.5%)	368 (52.3%)	147 (38.8%)	21 (6.7%)
Injection drug use (n, %)		0 (0%)	0 (0%)	0 (0%)	266 (85.0%)
Other/unknown (n, %)		38 (4.3%)	33 (4.7%)	38 (10.0%)	2 (0.6%)
Caucasians (n, %)		675 (77.5%)	474 (67.3%)	319 (84.2%)	289 (92.3%)
Diagnosed with HIV before 1 January 1995 (n, %)		132 (15.5%)	122 (17.3%)	89 (23.4%)	115 (36.7%)
Diagnosed with AIDS before start of HAART (n, %)		0 (0%)	311 (44.2%)	105 (27.7%)	58 (18.5%)
Viral load detectable 1 year after start of HAART (n, %)		0 (0%)	332 (47.2)	81 (21.4%)	105 (32.9%)
CD4 at diagnosis (median, IQR)		360 (240–513)	150 (50–370)	300 (120–504)	368 (230–550)
CD4 below 200 cells/ul 1 year after index date (n, %)		0 (0%)	297 (42.2%)	80 (21.1%)	63 (20.1%)
Started HAART after 1 January 2005 (n, %)		202 (23.2%)	153 (21.7%)	89 (23.5%)	45 (14.4%)

*IQR = interquartile range.

**: As patients could have more than one type of comorbidity, the sum of comorbidities is higher than the number of patients with comorbitiy.

The HIV population suffered from substantially increased mortality [MRR = 8.58 (95% confidence interval 5.79 to 12.71) among patients aged 25–45 and MRR = 6.00 (4.65 to 7.77)] among those aged 45–65). However, in Group 1 (HIV-infected patients without HIV risk factors, comorbidity, or alcohol/drug abuse) mortality was almost equal to that of the general population comparison cohort for the age group 45–65 [MRR = 1.14 (0.58 to 2.23)], but doubled for the age group 25–45 [MRR = 2.02 (0.61 to 6.70)] (Table 2).

**Table 2:** Mortality and mortality rate ratios by risk group.

Groups	Probability of survival from age 25 to 65 years	95% CI		Age interval	Deaths	PY**	MR***	95% confidence interval		MRR****	95% confidence interval	
Comparison cohort	0.88	0.86	0.90	25–45	37	29123	1.27	0.92	1.75			
				45–65	101	23899	4.23	3.48	5.14			
HIV overall	0.48	0.42	0.53	25–45	76	6973	10.90	8.70	13.64	8.58	5.79	12.71
				45–65	137	5395	25.39	21.47	30.02	6.00	4.65	7.77
Group 1*	0.86	0.77	0.92	25–45	4	2723	1.47	0.55	3.91	2.02	0.61	6.70
				45–65	11	1891	5.82	3.22	10.50	1.14	0.58	2.23
Group 2*	0.58	0.48	0.67	25–45	21	2525	8.32	5.42	12.76	4.62	2.48	8.60
				45–65	30	1772	16.93	11.84	24.21	4.27	2.57	7.08
Group 3*	0.30	0.21	0.40	25–45	15	826	18.15	10.94	30.11	12.75	4.64	35.09
				45–65	43	1032	41.68	30.91	56.20	10.79	6.29	18.52
Group 4*	0.03	0.003	0.12	25–45	36	898	40.07	28.90	55.55	32.60	12.79	83.08
				45–65	53	701	75.66	57.80	99.03	21.90	11.94	40.17

**Figure 1:**
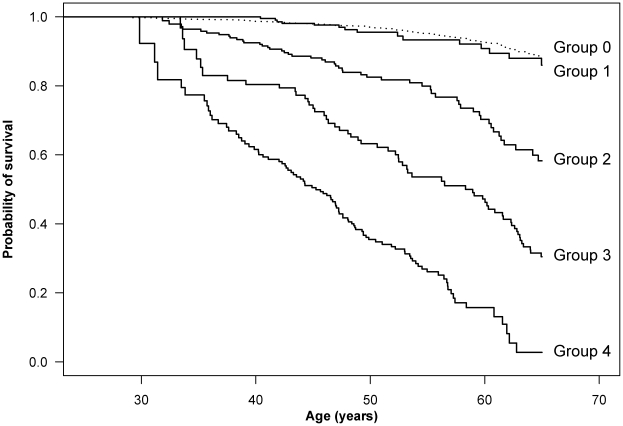
Cumulative survival for HIV-infected patients starting HAART and persons from the general population. Time was calculated from 1 year after start of HAART. The study population was categorized as: Group 0: Population comparison cohort (dotted line, N = 9,068). Group 1: HIV-infected patients without HIV risk factors, comorbidity or alcohol/drug abuse (N = 871). Group 2: HIV-infected patients with HIV risk factors, but no comorbidity or alcohol/drug abuse (N = 704). Group 3: HIV-infected patients with comorbidity, but no alcohol/drug abuse (N = 379). Group 4: HIV-infected patients with alcohol/drug abuse (N = 313). *HIV risk factors:* detectable viral load (>49 copies/ml) and/or CD4 below 200 cells/ul at the last measurement prior to the index date and/or AIDS- defining disease as of the index date. *Comorbidity:* diagnosed with comorbidity as defined in the Charlson Comorbidity Index before index date. *Abuse:* diagnosed with drug or alcohol abuse before index date or reporting drug abuse as route of HIV transmission.

*For definition of Groups 1–4, see Figure 1.

**PY = person-years.

***MR = mortality rate (deaths/1000 PY).

****MRR = mortality rate ratio.

The relative risk of death increased by risk group (Table 2), ranging from a fourfold increased risk of death in HIV patients aged 45–65 years with HIV risk factors, but no comorbidity or alcohol/drug abuse, to a more then 20-fold increased risk of death in HIV patients registered with alcohol or drug abuse. The probability of survival at 65 years of age was 0.48 among HIV-infected patients and 0.88 in the general population comparison cohort. However, for HIV patients with no risk factors, the probability of survival at age 65 was 0.86 (Figure 1). The probability of survival declined to 0.58 in Group 2 (patients with HIV risk factors), to 0.30 in Group 3 (HIV-infected patients with comorbidity), and to 0.03 in Group 4 (those with alcohol or drug abuse).

## Discussion

Our study confirms that HIV-infected patients on HAART suffer from a substantially increased risk of death compared to the general population. However, mortality was associated mainly with well-known HIV- and non-HIV-associated risk factors, which are identifiable prior to or in the initial phase of HAART treatment. Mortality in HIV-infected patients with no identifiable risk factors was almost identical to that of the general population with no risk factors. Importantly, increased risk of death was observed only in patients registered with one or more risk factors in the initial phase of HAART treatment.

The major strength of the study is its nationwide population-based design combined with long and nearly complete follow-up. Furthermore, access to comprehensive Danish medical and demographic databases permitted us to identify a well-matched population-based comparison cohort and allowed us to estimate the impact of major risk factors.

The study has several potential limitations. We could not account for smoking as we lacked information on tobacco use in the population comparison cohort. We relied on registry-based hospital diagnoses to identify comorbidities and alcohol/drug abuse and we had access to alcohol/drug abuse data for the comparison population only if it led to contact with the health care system. However, we assume that these factors cannot explain our results, as smoking and drug abuse is likely to be more prevalent among HIV-infected patients [16]. The risk-taking behavior leading to HIV acquisition also may have led to other exposures with long latency periods, not manifested at the index date. However, our findings do not indicate that such risk-taking behavior led to increased mortality in appropriately treated HIV-infected patients without other risk factors. More HIV patients were born outside Denmark which may give rise to some healthy survivor effects.

We included only the age group from 25–64 years, as the number of patients outside this range was too low to allow conclusions to be drawn. Therefore, our findings cannot be extended beyond this age interval. Few deaths were observed in the age group 25–45 years in the comparison cohort and in the HIV patients without risk factors why the estimates of risk of death in these categories are imprecise. Therefore we cannot rule out an increased risk of death in this age interval. In more than 60% of the HIV patients, one or more risk factors were identifiable why a substantial excess mortality is to be expected in the general HIV population.

It has been proposed that HIV is associated with premature aging, driven by residual inflammation, even with fully suppressed viral load. Although premature aging is not a well defined entity, it is thought to be associated with increased risk of death. As we did not observe substantially increased mortality among HIV patients without risk factors, our data does not support the theory of premature aging. Rather, the data establish that the increased risk of death in the HIV population on HAART mainly stems from classic risk factors.

Previous large-scale cohort studies comparing mortality in HIV-infected patients with that in the general population have found increased mortality in the HIV-infected group [1], [8], [17], [18]. However, several reports identified subsets of HIV-infected patients with no increased mortality compared with the general population (generally sexually infected patients on HAART with a CD4 cell count above 500 cells/mm^3^) [8], [17], [19]. In these reports the general population, in which a substantial amount of comorbidity and drug or alcohol abuse is present, was used as the reference. Therefore, the lack of association between HIV and mortality may have been due to confounding introduced by comorbidity, alcohol abuse and injection drug use in the general population. Because we used a comparison cohort from the general population restricted to persons with no comorbidity, alcohol or drug abuse, these confounders were eliminated from our study [20]. Our findings thus improve the level of evidence that HIV-infection, when treated optimally, does not increase the risk of death substantially.

Recently, comorbidity has been identified as the major risk factor for death among HIV-infected patients not yet on HAART [19]. We supplement this finding by demonstrating that comorbidity, as well as HIV risk factors and alcohol/drug abuse, are also major risk factors for death in HIV-infected patients on HAART.

We conclude that HIV-infected patients on HAART still suffer from substantial excess mortality, but the increased risk of death stems mainly from HIV- and non-HIV related risk factors, which can be identified prior to or in the initial phase of HAART treatment. Future management of the HIV-infected population should focus on early diagnosis, timely and effective HAART, and treatment of comorbidity and alcohol/drug abuse. Serious attention should be given to non-HIV related conditions among HIV-infected persons. However, stressing the impact of HIV on mortality after HAART initiation may severely hamper the patients' quality of life and be at odds with present data.
